# The High Burden of Malaria in Primary School Children in Southern Malawi

**DOI:** 10.4269/ajtmh.14-0618

**Published:** 2015-10-07

**Authors:** Don P. Mathanga, Katherine E. Halliday, Mpumulo Jawati, Allison Verney, Andrew Bauleni, John Sande, Doreen Ali, Rebecca Jones, Stefan Witek-McManus, Natalie Roschnik, Simon J. Brooker

**Affiliations:** Malaria Alert Centre, College of Medicine, University of Malawi, Blantyre, Malawi; London School of Hygiene and Tropical Medicine, London, United Kingdom; Save the Children International, Blantyre, Malawi; National Malaria Control Program, Ministry of Health, Lilongwe, Malawi; Save the Children USA, Washington, DC

## Abstract

Malaria among school children has received increased attention recently, yet there remain few detailed data on the health and educational burden of malaria, especially in southern Africa. This paper reports a survey among school children in 50 schools in Zomba District, Malawi. Children were assessed for *Plasmodium* infection, anemia, and nutritional status and took a battery of age-appropriate tests of attention, literacy, and numeracy. Overall, 60.0% of children were infected with *Plasmodium falciparum*, 32.4% were anemic and 32.4% reported sleeping under a mosquito net the previous night. Patterns of *P. falciparum* infection and anemia varied markedly by school. In multivariable analysis, higher odds of *P. falciparum* infection were associated with younger age and being stunted, whereas lower odds were associated with reported net use, higher parental education, and socioeconomic status. The odds of anemia were significantly associated with *P. falciparum* infection, with a dose–response relationship between density of infection and odds of anemia. No clear relationship was observed between health status and cognitive and educational outcomes. The high burden of malaria highlights the need to tackle malaria among school children.

## Introduction

Since the launch of Roll Back Malaria in 1998 there has been increased funding for malaria control and subsequent expansion in the coverage of malaria interventions.^1^,^2^ As a consequence, some areas of Africa have witnessed a marked decline in malaria transmission and disease burden.^3^–^7^ Notwithstanding this progress, an estimated 57% of the continent's population continues to live in areas of high malaria transmission where there has been little or no change in the burden of malaria between 2000 and 2010.^8^ One such country is Malawi where, despite the scale-up of malaria interventions, there has been limited decline in malaria transmission or hospital admissions over the last decade.^9^,^10^ The reasons for the lack of change are multifactorial, ranging from health system factors to the underlying high intensity of malaria transmission.^11^ In areas of high malaria transmission, modeling analysis shows that the breadth of coverage of malaria interventions across age groups needs to be broad to achieve substantive impact.^12^,^13^ Studies show that *Plasmodium* infection prevalence is typically highest among school-aged children^14^,^15^ yet this age group are often the least likely to be covered by malaria interventions^16^,^17^ and therefore represent an untreated reservoir of parasite transmission.^18^–^20^ The high rates of infection among school-aged children may also have a number of health and educational consequences for chronically infected children.^21^–^23^ To better understand the patterns of *Plasmodium* infection among school children in Malawi, we present analysis of health and education data from a cross-sectional survey conducted in 50 schools in Zomba District in southern Malawi.

## Materials and Methods

Reporting of this study has been verified in accordance with the Strengthening the Reporting of Observational Studies in Epidemiology (STROBE) checklist (Additional file 1).

### Study Setting.

**Figure 1.**
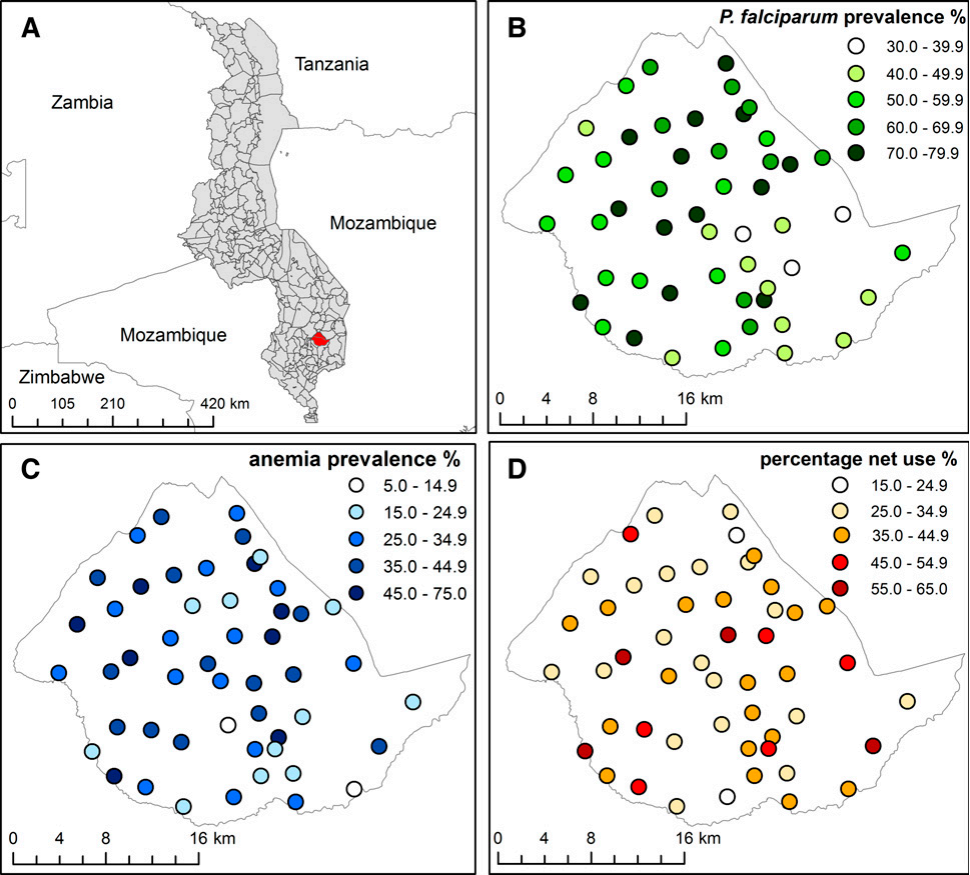
The location of TA Chikowi, Zomba District, in Malawi (**A**) and the geographical distribution of (**B**) *Plasmodium falciparum* infection, (**C**) anemia, and (**D**) reported mosquito net use (adjusted for age and sex) in 50 schools in TA Chikowi, Zomba District, southern Malawi, 2011.

This study was conducted in the area of Traditional Authority Chikowi in Zomba District, southern Malawi (Figure 1). In 2011 the region comprised 611 villages, with a population of approximately 206,081,^24^ served by 56 primary schools, with subsistence farming of maize and tobacco as the sources of income and employment. In Malawi, malaria remains the leading cause of morbidity and mortality,^25^ with an estimated 2.1 million cases per year in the 5–14 year age group.^26^ Transmission of malaria in the study area is intense, primarily because of the high average temperatures (17–27°C) and rainfall (1,144 mm).^27^ Despite the potential burden of malaria among these schools, no malaria programs specifically targeted at school children had been conducted.

The formal education system in Malawi comprises 8 years of primary education: classes 1–8 (known in Malawi as standards), 4 years of secondary and 4 years of university-level education. Primary school access has been free since the abolition of user fees in 1994. The 2010 Malawi Demographic Health Survey (DHS) indicated net attendance ratios (percentage of primary-school-age population (age 6–13) attending primary school) of 92% and 90% in females and males, respectively, despite a greater number of boys attending primary school than girls overall.^28^ Nationally, there is no substantial difference in attendance, grade repetition, or drop-out between sexes until age 15 years, when the attendance rate is substantially higher in males.^28^ Primary completion rate (access rate to class 8) is estimated to be 30–40% and reasons for drop-out include crowded classrooms, inadequate sanitary facilities, economic difficulties, and family responsibilities, some of which may impact more upon older girls than older boys.^29^ In Zomba District, where this study was conducted, the vast majority of schools (98.5%) are public, with only 5% of those offering facilities for boarding (Zomba District Education Office, personal communication). The pupil teacher ratio in Zomba is 76:1, similar to the national average of 74:1.^30^

### Ethical considerations.

The study was approved by the ethical review committees of the College of Medicine, Malawi (ref: P.02/11/1036) and LSHTM (ref: 5914). Prior to the study, stakeholders meetings were held at the national and district levels, following which, school-level sensitization meetings were held at each of the participating schools, where survey procedures and risks and benefits were explained and there was an opportunity to ask questions. Following participant selection, parents/guardians of randomly selected children were invited to a meeting with the study team at the school during which the selected parents were provided with an information sheet. They had an opportunity to seek further clarifications about the study and provide written informed consent. In addition, on the day of the survey, written assent was obtained from the children prior to their participation in the survey. The consent and assent procedures were conducted in the local language, Chichewa, and all information sheets and consent/assent forms were also written in Chichewa.

### Study Design.

**Figure 2.**
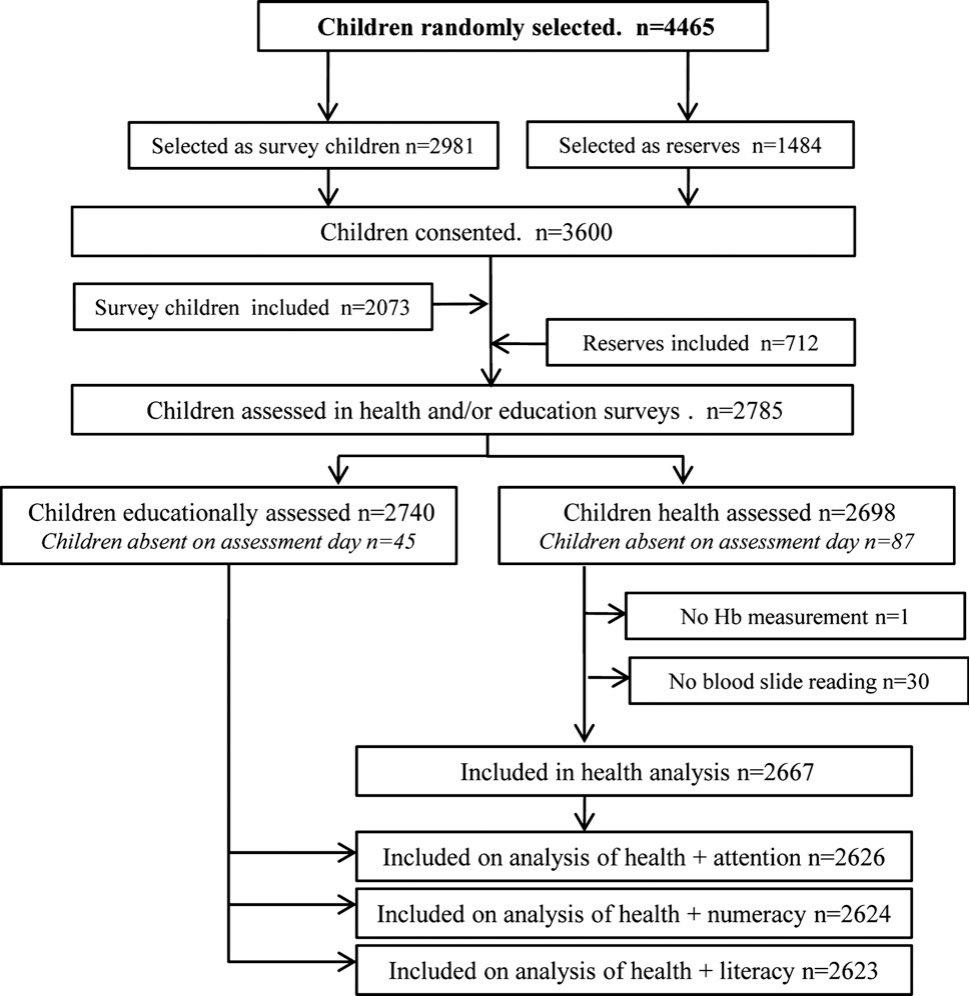
Data flow diagram for the education and health surveys conducted in 50 schools in TA Chikowi, Zomba District, southern Malawi, 2011.

Of the schools in TA Chikowi, 50 were randomly selected for inclusion in the study. The six remaining schools were selected to pilot the study tools and for future qualitative studies investigating the control of malaria in schools. Cross-sectional surveys of children enrolled in classes 1–8 of participating schools were carried out between March and April 2011. Prior to the survey, computer-generated random number tables were used to select four boys and four girls from each class to participate in the surveys, with two reserves of each gender selected in each class. These reserves were included if the selected student was not present on the day of the survey or the guardian did not provide consent (Figure 2). Where class sizes in a school were small, alternative classes in the same school were oversampled. Six schools comprised only lower classes (up to class 5, with the exception of one school which went up to class 3 only) and five schools did not have a class 8; in these schools no additional children were sampled.

### Health assessments.

For each child, axillary temperature was measured using a digital thermometer, and height and weight were measured to the nearest 0.1 cm and 0.1 kg using Leicester portable fixed base stadiometers and electronic balances, respectively. A finger-prick blood sample was collected from all children and used to prepare thin and thick blood smears for microscopy. The same finger prick sample was used to measure hemoglobin concentration by a portable hemoglobinometer (Hemocue, Ängelholm, Sweden). Children with an axillary temperature of 37.5°C or greater were tested with a malaria rapid diagnostic test (RDT; SD Bioline Ag Pf/Pan^®^ RDT) and those found positive for *Plasmodium* parasitemia were treated with artemether–lumefantrine as per national guidelines. Children with a hemoglobin concentration < 80 g/L were provided with ferrous sulfate and albendazole.

Blood slides were stained with a 3% Giemsa solution for 45 minutes. The number of asexual forms of *Plasmodium* per 200 white blood cells (WBCs) was counted and parasite densities were computed assuming a mean WBC count of 8,000 cells/μL. Slides were declared negative if no parasites were found after examining 100 high-power fields. Slide reading was blinded and slides were read in duplicate at the Malaria Alert Center reference laboratory in Blantyre and all discrepant results were resolved based on a third reading.

### Educational assessments.

Children were assessed using age-appropriate tests of sustained attention, literacy, and numeracy. All assessment tools were piloted prior to use to assess their cultural and class-appropriate validity and subsequently adapted for the Malawian context. The assessments were administered to pairs of classes (1–2, 3–4, 5–6, and 7–8) and administered at the individual level in classes 1 and 2 and at the group level for all other classes. Education assessments were conducted between 2 and 5 days prior to the health assessments so that the real-time effect of the health status of the children on their cognitive and educational performance could be assessed, without being affected by potential treatment during the health surveys.

The pencil tap task was used to assess sustained attention and executive function among children in classes 1 and 2, who were required to tap a pencil on a desk a predetermined number of times in response to the assessor's tap while simultaneously completing a shading task.^31^ The code transmission test, adapted from the tests of everyday attention for children (TEA–Ch tests),^32^ was used to assess sustained attention in the upper classes. The assessment involves listening to a prerecorded list of digits read out at a standardized speed and children are required to listen out for a “code”—two consecutive occurrences of the number 5—and then record the one or two (known as the 1-and 2-number task, respectively) numbers that preceded the code. The 1-number task was administered to classes 3 and 4 while the 2-number task was administered to classes 5–8. Numeracy was assessed through a number identification and quantity discrimination task in classes 1 and 2, a missing number and arithmetic task in classes 3 and 4, both of which were adapted from the Early Grade Mathematics Assessment,^33^ and a written arithmetic assessment in classes 5–8. In regards to literacy, classes 1 and 2 were assessed using letter identification adapted from the Early Grade Reading Assessment^34^ and for classes 3–8 spelling tasks were adapted from the Phonological Awareness Literacy Screening tool,^35^ whereby credit was given for phonologically acceptable spellings in the lower classes (3 and 4) and correctly identified features and sound combinations of the words in the higher classes.

### Sociodemographic indicators.

Parents of study children were requested to attend the school on the day of the health assessments to observe procedures, collect treatment for the child if required, and to complete a questionnaire relating to family size, ownership of possessions, their education level, and mosquito net use by themselves and their children. Only 146 parents did not attend to observe the surveys and hence were unable to provide data on household characteristics. This remarkable attendance rate is likely because of the excellent community relationships which Save the Children has with the local communities. Interviews were also conducted with head teachers of each school to ascertain school size, enrollment numbers, and school health programs such as school feeding and deworming. Schools were geolocated using a global positioning system receiver (eTrex Garmin Ltd., Olathe, KS).

### Definitions.

Malaria parasitemia was defined as the presence of *Plasmodium* parasites in the blood identified by expert microscopy. Clinical malaria was defined as axillary temperature ≥ 37.5°C and a positive blood slide. Anemia was defined according to World Health Organization age- and sex-specific thresholds of hemoglobin concentration < 110 g/L in children under 5 years; < 115 g/L in children aged 5–11 years; < 120 g/L in females aged 12 years and over and males aged 12–15 years; and < 130 g/L in males over 15 years, without adjustment for altitude.^36^ Anthropometric indices were calculated using Stata software (Stata Corporation, College Station, TX), assuming a mid-year age for each child.^37^ Children were classified as stunted, underweight or thin if z–scores for height-for-age, weight-for-age, and body mass index were less than two standard deviations (SD) below the respective reference median. Asset ownership was used to derive an index of socioeconomic status (SES) using principal component analysis.^38^ Ownership of items such as a bicycle, motorcycle, radio, television, and mobile phone, as well as household construction and facilities variables were all included, with the first principal component explaining 25% of the variation.

### Data analysis.

Data were double-entered in Microsoft Access (Microsoft Corporation, Seattle, WA), linked by school location and visualized using ArcGIS 10 (Environmental Systems Research Institute Inc., Redlands, CA) and imported into Stata v.12 software for statistical analysis. Prevalence estimates are presented with 95% binomial confidence intervals (CIs) and continuous variables are summarized using arithmetic means and SD. For both health and educational outcomes, risk factors with associations at the 10% significance level in a univariable model were subsequently included in a multivariable model, and stepwise elimination was used to create minimum adequate models using a 5% significance level for retention. All statistical analysis was performed using mixed effects linear or logistic regression models with a random effect of school to account for correlations between children within schools. Scores for the three cognitive and education outcomes (sustained attention, literacy, and numeracy) were standardized separately by class, permitting combined statistical analysis across classes that received different versions of the tests and thereby simultaneously controlling for both class and test version. Non normality of the cognitive and educational scores was addressed through bootstrapping, whereby schools were resampled to account for school-level clustering.^39^ Analysis was conducted for the lower classes (1–4) and the higher classes (5–8) separately, to allow the influence of the characteristics of interest to vary between the younger and older children.

## Results

### Study population characteristics.

Of the 3,600 school children from the 50 schools for whom consent was provided, 2,785 participated in either health and/or education surveys (Figure 2). There were no specific refusals to participate or withdrawals by either parents or children, non-participation at any stage was due to absence of the parent at the consent meetings or the child on the assessment days. A total of 2,667 children were included in the health analysis and 2,623 children were included in the education and health analysis (Figure 2). The age range of children was 5–21 years, but 97.6% were under 18 years of age and the mean was 11.8 years (SD 3.1) with 47.7% of participants being male (Table 1). The overall prevalence of *P. falciparum* infection was 60.0% (95% CI: 56.3–63.8%). No other *Plasmodium* species were detected. The majority of infections were light, with 73.8% of those infected having parasite densities below 1,000 cells/μL. Fever in the last 2 weeks was reported by 967 children (36.6%), but the prevalence of fever (as measured by a digital thermometer) on the day of the health surveys was low, with only 2.6% of children with a temperature of ≥ 37.5°C, and of those 76 children, 50 had a positive blood slide.

The mean hemoglobin was 125.1 g/L (SD 15.6 g/L) and the prevalence of anemia was 32.4% (95% CI: 29.3–35.5%). Both *P. falciparum* infection and anemia exhibited marked variation by school: 32.5–83.9% and 5.4–72.7%, respectively (Figure 1). No clear patterns by sex were observed for either *P. falciparum* infection or anemia, although both declined with increasing age. The overall prevalence of stunting, underweight, and thinness was 29.0%, 17.6% and 6.4%, respectively. In total, 69.6% of households were reported as possessing at least one bed net; however, only 38.2% children reported usually sleeping under a net and 32.4% as having slept under a net the previous night with girls more likely reporting net use than boys (*P* = 0.047).

### Predictors of *Plasmodium* infection.

As shown in Table 1, in both univariable analysis, and after controlling for all significantly associated variables, increasing age, mosquito net use the previous night, having three or more nets in the house, secondary or tertiary education for the parent and higher SES were associated with reduced odds of *P. falciparum* infection. Surprisingly, child-reported fever in the last two weeks was also associated with reduced odds of infection, although child-reported fever on the day of assessment was not associated. Stunting was associated with increased odds of *P. falciparum* infection. No significant difference in odds of *P. falciparum* infection was observed between sexes

### Predictors of anemia.

In univariable analysis, the odds of anemia were reduced with increasing age, higher SES and high education level of the parent (Table 2). On the other hand, increased numbers of people living in the household and indicators of nutritional status (stunting, being thin, or underweight), were associated with increased odds of anemia. Infection with *P. falciparum* parasites was significantly associated with increased odds of anemia, with a positive dose–response relationship observed between density of infection and odds of anemia. Children with low-density infections had over one and half times the odds of being anemic and those with medium-/high-density infections (≥ 1,000 cells/μL) had greater than two and a half times the odds of being anemic. In multivariable analysis, once controlling for other related variables, increasing age, stunting, thinness, and *P. falciparum* infection remained significantly associated with anemia (Table 2).

### Predictors of cognitive and educational performance.

Table 3 presents the results of the univariable associations between individual, household, and school-level factors and standardized scores of sustained attention, numeracy, and literacy for younger children (classes 1–4), and Table 4 presents the same for older children (classes 5–8). The multivariable analyses for both younger and older classes are shown in Table 5. Due to class-based standardization of scores, results from both the univariable and multivariable models are tacitly adjusting for class and test.

When considering the relationship between health status and cognitive and educational performance, no association was observed between *Plasmodium* infection and scores of sustained attention, numeracy, or literacy for either the younger or older classes. There was likewise no evidence of a relationship between anemia and either sustained attention or numeracy. However, somewhat surprisingly anemia was associated with improved performance in literacy assessments in the older (but not the younger) classes in both unadjusted and adjusted analysis (Table 5). On the other hand, analyses showed indicators of nutritional status (stunting and thinness) to be associated with poorer performance on numeracy assessments in the younger (but not the older) classes (Table 5).

In relation to demographic factors, performance on numeracy in the younger classes improved with increasing age within the class, whereas in the older classes increasing age was associated with poorer performance on tests of both sustained attention and literacy within the class (Table 5). Also, for sustained attention and literacy in the higher classes, girls were found to perform significantly worse than boys, between 0.1 and 0.2 SD below the class mean. With respect to household correlates, weak evidence was observed for univariable associations between children living in households where the parent had some secondary education and with higher SES and better performance in literacy assessments in the lower classes (Table 3); however, these associations were not retained in adjusted analysis. In the older classes, a greater number of people in the household were associated with lower literacy and more children per household with poorer performance in sustained attention (Table 5). Finally, when considering school-level characteristics, despite weak evidence of a relationship between increased pupil teacher ratios and poorer sustained attention and literacy scores in the older classes (Table 4), these associations did not hold once adjusting for additional characteristics.

## Discussion

Effective malaria control must be based on appropriate information about the malaria burden faced by populations and the needs of specific subgroups. Epidemiological data on the burden of malaria among school populations are few, with most studies conducted in east Africa^21^,^40^–^42^ and West Africa.^43^,^44^ Data for southern Africa are particularly scant: for example, the Malaria Atlas Project, which provides maps and estimates to guide planning of malaria control, includes only six surveys conducted among school-aged children conducted between 1985 and 1997 in Mozambique, Swaziland, and Zambia.^45^ The prevalence of *P. falciparum* found in the present study (60%) is substantially higher than estimates in these studies elsewhere in the region and higher than the prevalence of infection among under 5s in southern Malawi in the 2012 Malaria Indicator Survey.^46^ Until recently, school children were neglected by the malaria control program in Malawi where insecticide-treated nets (ITNs) and other interventions were mainly targeted at under 5s and pregnant women. Only in the most recent Malawi Malaria Strategic Plan (2011–2015) have efforts been made to provide universal coverage of ITNs, specifically citing schools as a key delivery platform.^47^

Our study also found a high prevalence of anemia (32.4%) which showed a strong positive relationship with *P. falciparum* infection. Such a finding adds to the growing body of evidence on the hematological consequences of malaria parasitemia among African school children.^40^,^48^–^50^ The exact mechanisms by which chronic parasitemia causes anemia are multifactorial and include direct destruction of the erythrocytes by the parasite and the pro-inflammatory mediator response during the blood stage of infection^51^ and disruption of erythropoiesis in the bone marrow^51^; although the relative role of different mechanisms is unclear. What is clear is that effective control of malaria among school children can lead to marked improvements in hemoglobin concentration.^52^,^53^

As one would expect, the use of ITNs was associated with reduced risk of *P. falciparum* infection. However, only 32.4% of the school children had slept under a net the previous night, which is similar to the national average of children aged 5–14 years (30.2%), but substantially lower than the national average for children under 5 years of age (56%) and other older household members^46^—a finding demonstrated elsewhere in Africa.^16^ Efforts should be made to educate school children on the importance of ITN use and to further understand why net utilization remains low in this age group when, through mass campaigns, many countries in the region are providing an ITN for every two household members to achieve universal coverage.^54^

Among the study children, stunting was a strong risk factor for both *P. falciparum* infection and anemia. Chronic malnutrition has been known to increase the risk of infection of malaria, and children who are already chronically malnourished generally have lower hemoglobin levels to begin with,^48^ exaggerating the effects of malaria and the resulting anemia.^55^,^56^ Although the presence of a school feeding program was not significantly associated with reduced risk of anemia in this study, an association that has been found elsewhere.^40^,^57^ At the time of the study only a few schools in the area had a school feeding program.

In contrast to the relationships between *P. falciparum* infection and anemia, the relationships between malaria and cognition and educational achievement are more complex. The observed age and sex differences in sustained attention and literacy are consistent with a previous study on the Kenyan coast which use the same educational assessment tools.^40^ Such differentials are considered to reflect gender differences in access to education and increased grade repetition.^58^–^60^ The strongest evidence indicating malaria impairs cognition and educational achievement comes from intervention trials. In a cluster-randomized, double-blind placebo-controlled trial conducted in western Kenya, intermittent preventive treatment (IPT) with sulfadoxine–pyrimethamine in combination with amodiaquine significantly improved sustained attention of 10–12-year-old schoolchildren.^53^ In Sri Lanka, a randomized, placebo-controlled, double-blind trial of chloroquine prophylaxis in children aged 6–12 years showed that educational attainment improved and that school absenteeism was reduced significantly in children who took malaria prophylaxis.^43^ Associations between malaria parasitemia and cognitive function have additionally been demonstrated in cross-sectional surveys in Uganda,^21^ Mali,^61^ and Zambia.^62^ By contrast, no association between malaria and cognition was found in a low-moderate transmission setting on the south coast of Kenya.^40^ Possible reasons for the differing findings of studies include variation in the underlying intensity of malaria transmission and density of parasitemia in those infected and the influence of socioeconomic factors on cognition and education.^63^,^64^

This survey used a robust sampling design to assess the burden of malaria and anemia among children present in school on the day of the survey. However, the nature of the study did not consider non-enrolled children or children absent on the day of the survey; such children may be less healthy and more vulnerable to malaria and anemia than those present in school. The low school completion rate related to social and economic factors such as household wealth and family responsibilities (e.g., the requirement to assist at home), and the interaction of these with gender, may have obscured a true relationship between malaria, anemia, cognitive outcomes, and sociodemographic factors, particularly in the higher classes. A second limitation is that household data were self-reported by parents and children and were not validated by home-based visits and observations, and as such, results may be subject to recall and information bias.

In conclusion, these results demonstrate the high burden of malaria and anemia among school-going children in Zomba District, Malawi. Hence there is a need for school-based interventions for malaria control since schools provide a natural access point for school-going children and such school-based interventions have been shown to be effective in reducing malaria morbidity and mortality.^43^,^52^,^53^,^65^ Such interventions, if well integrated into the existing school health programs, could ensure the goal of universal access to effective malaria interventions and enable school children to fulfill their optimum learning potential.

## Figures and Tables

**Table 1:** Predictors of *Plasmodium falciparum* infection among school children in Zomba District, Malawi in 2011

Variable*	No. (%) of children† (*N* = 2,667)	No. (%) children with *P. falciparum* (*N* = 1,601)	Univariable analysis		Multivariable analysis	
			OR (95% CI)	*P* value‡	Adjusted OR (95% CI) (*N* = 2,364)	*P* value‡
Sex						
Male	1,271 (47.7)	780 (61.4)	Reference			
Female	1,396 (52.3)	821 (58.8)	0.90 (0.76, 1.05)	0.179		
Age (per additional year)	11.81 (3.15)	−	0.93 (0.91, 0.96)	< 0.001		
Age (years)						
5–9	660 (24.7)	420 (63.6)	Reference		Reference	
10–12	1,125 (42.2)	724 (64.4)	1.02 (0.83, 1.26)		0.98 (0.79, 1.23)	
13–18	882 (33.1)	457 (51.8)	0.60 (0.48, 0.74)	< 0.001	0.55 (0.43, 0.69)	< 0.001
WAZ§∥						
Not underweight	681 (82.4)	436 (64.0)	Reference			
Underweight	146 (17.6)	96 (65.8)	1.09 (0.73, 1.63)	0.661		
HAZ§						
Not stunted	1,881 (71.0)	1,093 (58.1)	Reference		Reference	
Stunted	768 (29.0)	449 (65.0)	1.33 (1.11, 1.60)	0.002	1.33 (1.09, 1.62)	0.004
BMIZ§						
Not thin	2,480 (93.6)	1,488 (60.0)	Reference			
Thin	169 (6.4)	104 (61.5)	1.08 (0.78, 1.51)	0.633		
De-wormed						
No	526 (21.2)	310 (58.9)	Reference			
Yes	1,958 (78.8)	1,187(60.6)	1.09 (0.89, 1.34)	0.420		
Net use last night (child reported)						
No	1,798 (67.6)	1,107 (61.6)	Reference		Reference	
Yes	860 (32.4)	487 (56.6)	0.83 (0.70, 0.99)	0.034	0.79 (0.66, 0.96)	0.017
Reported fever in last 2 weeks						
No	1,678 (63.4)	1,046 (62.3)	Reference		Reference	
Yes	967 (36.6)	541 (56.0)	0.81 (0.68, 0.96)	0.016	0.79 (0.66, 0.95)	0.011
Reported fever now						
No	1,905 (72.1)	1,134 (59.5)	Reference			
Yes	736 (27.9)	449 (61.0)	1.08 (0.90, 1.30)	0.390		
Household level						
Education of household head						
No education	440 (17.5)	273 (62.1)	Reference		Reference	
Some/completed primary	1,755 (69.6)	1,064 (60.6)	0.91 (0.73, 1.13)		0.89 (0.70, 1.14)	
Some/completed secondary	326 (12.9)	173 (53.1)	0.63 (0.47, 0.86)	0.007	0.65 (0.46, 0.92)	0.036
Socioeconomic status						
Poorest	494 (20.2)	293 (59.3)	Reference		Reference	
Poor	501 (20.4)	302 (60.3)	1.10 (0.85, 1.43)		1.08 (0.82, 1.42)	
Medium	527 (21.5)	349 (66.2)	1.31 (1.00, 1.71)	< 0.001	1.30 (0.98, 1.72)	< 0.001
Less poor	445 (18.2)	279 (62.7)	1.15 (0.88, 1.51)		1.26 (0.94, 1.70)	
Least poor	484 (19.7)	239 (49.4)	0.67 (0.52, 0.88)		0.74 (0.55, 1.00)	
Number of people in house	5.28 (2.27)	−	1.02 (0.98–1.06)	0.360		
Number of nets in house						
No nets	747 (30.4)	456 (61.0)	Reference		Reference	
1–2 nets	1,161 (47.3)	729 (62.8)	1.14 (0.93–1.39)		1.12 (0.91, 1.39)	
≥ 3 nets	546 (22.3)	283 (51.8)	0.74 (0.58–0.93)	< 0.001	0.83 (0.64, 1.08)	0.036
School level						
School feeding						
No	2,283 (85.6)	1,385 (60.7)	Reference			
Yes	384 (14.4)	216 (56.3)	0.85 (0.55, 1.31)	0.449		

BMI = body mass index for age; CI = confidence interval; HAZ = height for age; OR = odds ratio; SES = socioeconomic status; WAZ = weight for age.

* Displayed as number and percentage except for continuous variables, displayed as mean and standard deviation (SD).

† All characteristics have less than 2% missing data with the exception of following indicators: WAZ, deworming (7.0% missing), education level of household head (4.5% missing) SES, and reported net use (8.0% missing).

‡ *P* value is from likelihood ratio test comparing multilevel logistic regression models (accounting for school level clustering), with and without the factor of interest.

§ Underweight, stunted and thin defined as WAZ, HAZ, and BMIZ z-scores < 2 SD.

∥ WAZ only calculated for children aged 5–10 years (thus only presented for 827 children).

**Table 2:** Predictors of anemia among school children in Zomba District, Malawi in 2011

Variable	No. (%) Children with Anemia (*N* = 864)	Univariable analysis		Multivariable analysis	
		OR (95% CI)	*P* value*	Adjusted OR (95% CI) (*N* = 2,504)	*P* value*
Sex					
Male	411 (32.3)	Reference			
Female	453 (32.4)	1.00 (0.84, 1.18)	0.975		
Age (per additional year)		0.92 (0.90, 0.94)	< 0.001	0.92 (0.89, 0.95)	< 0.001
Age (years)					
5–9	278 (41.2)	Reference			
10–12	337 (30.0)	0.59 (0.48, 0.72)			
13–18	249 (28.2)	0.54 (0.43, 0.67)	< 0.001		
*Plasmodium falciparum* infection†					
No	276 (25.9)	Reference		Reference	
Yes	588 (36.7)	1.68 (1.41, 2.01)	< 0.001	1.58 (1.31, 1.91)	< 0.001
Parasite density (p/μL)†					
No infection (0)	276 (25.9)	Reference			
Low (1–999)	353 (35.1)	1.59 (1.31, 1.94)			
Medium (> 1,000)	169 (47.2)	2.57 (1.98, 3.33)	< 0.001		
WAZ‡§					
Not underweight	253 (37.2)	Reference			
Underweight	67 (45.9)	1.43 (0.98, 2.09)	0.062		
HAZ‡					
Not stunted	566 (30.1)	Reference		Reference	
Stunted	294 (38.3)	1.46 (1.22, 1.75)	< 0.001	1.48 (1.22, 1.80)	< 0.001
BMIZ‡					
Not thin	792 (31.9)	Reference		Reference	
Thin	68 (40.2)	1.45 (1.04, 2.02)	0.029	1.42 (1.01, 2.02)	0.046
De-wormed					
No	178 (33.8)	Reference			
Yes	645 (32.9)	0.95 (0.77, 1.17)	0.631		
Fever in last 2 weeks					
No	539 (32.1)	Reference			
Yes	316 (32.7)	1.05 (0.87, 1.27)	0.529		
Household level					
Education of household head					
No education	131 (29.8)	Reference			
Some/completed primary	607 (34.6)	1.22 (0.97, 1.54)			
Some/completed secondary	96 (29.4)	0.91 (0.66, 1.26)	0.037		
Socioeconomic status					
Poorest	172 (34.8)	Reference			
Poor	176 (35.1)	1.02 (0.78, 1.33)			
Medium	183 (34.7)	1.00 (0.77, 1.31)	0.056		
Less poor	144 (32.4)	0.88 (0.67, 1.16)			
Least poor	136 (28.1)	0.71 (0.54, 0.94)			
Number of people in house	−	1.03 (0.99–1.08)	0.094		
School level					
School feeding program					
No	766 (33.6)	Reference			
Yes	98 (25.5)	0.72 (0.47, 1.08)	0.121		

OR = odds ratio; BMIZ = body mass index for age; CI = confidence interval; HAZ = height for age; OR = odds ratio; WAZ = weight for age.

* *P* value is from likelihood ratio test comparing multilevel logistic regression models (accounting for school level clustering), with and without the factor of interest.

† Binary *Plasmodium* infection variable and categorical infection density variable collinear so only infection status variable included in the multivariable model.

‡ Underweight, stunted and thin defined as WAZ, HAZ, and BMIZ z-scores < 2 SD.

§ WAZ only calculated for children aged 5–10 years. Therefore, not included in multivariable model.

**Table 3:** Univariable associations between individual, household and school-level risk factors, and standardized scores of sustained attention, numeracy and literacy in children from classes 1 to 4, in Zomba District, Malawi in 2011

Variable	Sustained attention		Numeracy		Literacy	
	Mean difference in standardized scores*	*P* value†	Mean difference in standardized scores*	*P* value†	Mean difference in standardized scores*	*P* value†
Child level						
Sex						
Male	Reference		Reference		Reference	
Female	−0.13 (−0.27, −0.00)	0.062	−0.05 (−0.14, 0.05)	0.271	−0.01 (−0.15, 0.11)	0.926
Age (per additional year)‡	0.03 (−0.00, 0.06)	0.078	0.03 (0.00, 0.06)	0.040	0.01 (−0.01, 0.03)	0.395
*Plasmodium falciparum* infection*						
No	Reference		Reference		Reference	
Yes	0.04 (−0.11, 0.19)	0.605	0.03 (−0.10, 0.15)	0.697	−0.00 (−0.12, 0.10)	0.960
Parasite density (p/μL)*						
No infection (0)	Reference		Reference		Reference	
Low (1–999)	0.04 (−0.11, 0.19)	0.820	0.07 (−0.07, 0.22)	0.174	0.03 (−0.09, 0.16)	0.505
Medium/high (> 1,000)	0.04 (−0.10, 0.20)		−0.03 (−0.18, 0.11)		−0.07 (−0.21, 0.08)	
Anemia status						
Not anemic	Reference		Reference		Reference	
Anemic	−0.04 (−0.14, 0.07)	0.492	−0.03 (−0.17, 0.12)	0.694	−0.03 (−0.13, 0.07)	0.599
HAZ§						
Not stunted	Reference		Reference		Reference	
Stunted	0.01 (−0.09, 0.12)	0.814	−0.11 (−0.22, 0.01)	0.065	−0.03 (−0.13, 0.09)	0.626
BMIZ§						
Not thin	Reference		Reference		Reference	
Thin	−0.12 (−0.36, 0.11)	0.313	−0.19 (−0.40, −0.00)	0.056	−0.04 (−0.27, 0.17)	0.709
De-wormed						
No	Reference		Reference		Reference	
Yes	−0.05 (−0.19, 0.08)	0.442	0.03 (−0.08, 0.16)	0.603	0.03 (−0.13, 0.16)	0.619
Household level						
Education of household head						
No education	Reference		Reference		Reference	
Some/completed primary	−0.10 (−0.25, 0.06)	0.381	0.01 (−0.15, 0.10)	0.923	−0.05 (−0.16, 0.05)	0.098
Some/completed secondary	−0.04 (−0.21, 0.13)		0.04 (−0.15, 0.21)		0.10 (−0.06, 0.27)	
Socioeconomic status						
Poorest	Reference		Reference		Reference	
Poor	0.01 (−0.15, 0.18)		0.03 (0.16, 0.23)	0.642	0.24 (0.06, 0.43)	
Median	0.02 (−0.14, 0.19)	0.722	0.12 (−0.05, 0.32)	0.191	0.21 (0.03, 0.40)	0.075
Less poor	−0.07 (−0.23, 0.09)		0.04 (−0.17, 0.23)		0.12 (−0.06, 0.28)	
Least poor	−0.04 (−0.21, 0.16)		0.02 (−0.15, 0.21)		0.06 (−0.09, 0.23)	
Number people in house‡						
	0.01 (−0.01, 0.02)	0.233	0.00 (−0.01, 0.04)	0.790	0.01 (−0.01, 0.04)	0.460
Number children in house‡						
	0.01 (−0.02, 0.05)	0.366	0.02 (−0.02, 0.05)	0.250	0.02 (−0.01, 0.05)	0.213
School level						
School feeding program						
No	Reference		Reference		Reference	
Yes	0.17 (−0.09, 0.39)	0.190	−0.11 (−0.32, 0.11)	0.337	0.02 (−0.23, 0.27)	0.847
Pupil:teacher						
< 50.0	Reference		Reference		Reference	
50.0–99.9	0.16 (−0.05, 0.36)	0.213	−0.24 (−0.54, 0.03)	0.109	0.01 (−0.28, 0.33)	0.289
≥ 100.0	0.03 (−0.19, 0.25)		−0.27 (−0.57, −0.04)		−0.15 (−0.43, 0.14)	

BMIZ = body mass index for age; CI = confidence interval; HA = height for age; SD = standard deviation.

95% CI are obtained from bias-corrected bootstrap analyses using 2,000 bootstrap samples.

* Scores standardized separately by class for each of the three domains (attention, numeracy and literacy), so as to control for the effect of class and test. Differences are presented as z-scores from the class mean.

† *P* value is from the Wald test using multilevel linear regression models (accounting for school level clustering).

‡ Modeled as a continuous variable.

§ Stunted and thin defined as HAZ and BMIZ z-scores < 2 SD.

**Table 4:** Univariable associations between individual, household and school-level risk factors, and standardized scores of sustained attention, numeracy and literacy in children from classes 5 to 8, in Zomba District, Malawi in 2011

Variable	Sustained attention		Numeracy		Literacy	
	Mean difference in standardized scores*	*P* value†	Mean difference in standardized scores*	*P* value†	Mean difference in standardized scores*	*P* value†
Child level						
Sex						
Male	Reference		Reference		Reference	
Female	−0.10 (−0.22, 0.02)	0.094	−0.09 (−0.20, 0.02)	0.126	−0.14 (−0.25, −0.04)	0.007
Age (per additional year)‡						
	−0.02 (−0.05, 0.00)	0.078	−0.00 (−0.03, 0.02)	0.712	−0.06 (−0.09, −0.03)	< 0.001
*Plasmodium falciparum* infection*						
No	Reference		Reference		Reference	
Yes	0.03 (−0.07, 0.13)	0.543	−0.03 (−0.15, 0.08)	0.572	0.00 (−0.07, 0.07)	0.669
Parasite density (p/μL)*						
No infection (0)	Reference		Reference		Reference	
Low (1–999)	0.08 (−0.04, 0.21)	0.264	0.02 (−0.10, 0.14)	0.276	0.07 (−0.03, 0.17)	0.279
Medium/high (> 1,000)	−0.06 (−0.23, 0.11)		−0.18 (−0.43, 0.05)		0.10 (−0.09, 0.30)	
Anemia status						
Not anemic	Reference		Reference		Reference	
Anemic	−0.01 (−0.14, 0.13)	0.826	−0.04 (−0.14, 0.08)	0.548	0.12 (0.01, 0.22)	0.024
HAZ§						
Not stunted	Reference		Reference		Reference	
Stunted	0.06 (−0.07, 0.20)	0.412	0.02 (−0.09, 0.13)	0.714	0.01 (−0.09, 0.12)	0.790
BMIZ§						
Not thin	Reference		Reference		Reference	
Thin	0.11 (−0.07, 0.29)	0.245	−0.04 (−0.26, 0.21)	0.730	0.10 (−0.08, 0.31)	0.296
De-wormed						
No	Reference		Reference		Reference	
Yes	−0.09 (−0.22, 0.05)	0.181	−0.09 (−0.23, 0.05)	0.239	0.03 (−0.13, 0.20)	0.674
Household level						
Education of household head						
No education	Reference		Reference		Reference	
Some/completed primary	−0.08 (−0.24, 0.09)	0.601	−0.02 (−0.15, 0.10)	0.650	0.03 (−0.11, 0.16)	0.849
Some/completed secondary	−0.11 (−0.35, 0.11)		−0.08 (−0.27, 0.12)		0.06 (−0.16, 0.29)	
Socioeconomic status						
Poorest	Reference		Reference		Reference	
Poor	−0.10 (−0.27, 0.07)		−0.10 (−0.29, 0.08)		−0.07 (−0.25, 0.11)	
Median	0.08 (−0.07, 0.23)	0.241	−0.18 (−0.33, −0.03)	0.191	0.07 (−0.08, 0.24)	0.572
Less poor	0.05 (−0.13, 0.24)		−0.11 (−0.31, 0.08)		0.05 (−0.16, 0.25)	
Least poor	−0.00 (−0.20, 0.19)		−0.09 (−0.26, 0.07)		0.04 (−0.14, 0.21)	
Number of people in house‡	−0.02 (−0.05, 0.00)	0.058	−0.03 (−0.05, −0.00)	0.030	−0.02 (−0.05, 0.00)	0.070
Number of children in house‡	−0.04 (−0.06, −0.01)	0.006	−0.02 (−0.05, 0.00)	0.244	−0.01 (−0.04, 0.01)	0.560
School level						
School feeding program						
No	Reference		Reference		Reference	
Yes	0.12 (−0.03, 0.29)	0.152	0.20 (−0.21, 0.62)	0.351	0.23 (−0.09, 0.55)	0.151
Pupil:teacher						
< 50.0	Reference		Reference		Reference	
50.0–99.9	−0.20 (−0.50, 0.04)	0.098	−0.41 (−1.43, 0.12)	0.581	−0.30 (−0.74, −0.00)	0.088
≥ 100.0	−0.36 (−0.74, −0.06)		−0.40 (−1.39, 0.16)		−0.42 (−0.84, −0.10)	

CI = confidence interval; BMIZ = body mass index for age; HA = height for age; SD = standard deviation.

95% CI are obtained from bias-corrected bootstrap analyses using 2,000 bootstrap samples.

* Scores standardized separately by class for each of the three domains (attention, numeracy, and literacy), so as to control for the effect of class and test. Differences are presented as z-scores from the class mean.

† *P* value is from the Wald test using multilevel linear regression models (accounting for school-level clustering).

‡ Modeled as a continuous variable.

§ Stunted and thin defined as HAZ and BMIZ z-scores < 2 SD.

**Table 5:** Multivariable associations between individual and household risk factors and standardized scores of numeracy in children from classes 1 to 4, and in sustained attention, numeracy and literacy in children from classes 5 to 8, in Zomba, Malawi in 2011

Variable	Numeracy (classes 1–4)		Sustained attention (classes 5–8)		Numeracy (classes 5–8)		Literacy (classes 5–8)	
	Mean difference in standardized scores	*P* value†	Mean difference in standardized scores*	*P* value†	Mean difference in standardized scores*	*P* value†	Mean difference in standardized scores‡	*P* value†
Child level								
Sex								
Male			Reference				Reference	
Female			−0.14 (−0.26, −0.01)	0.025			−0.18 (−0.29, −0.07)	0.001
Age (per year)‡	0.04 (0.01, 0.08)	0.011	−0.03 (−0.05, −0.01)	0.019			−0.07 (−0.10, −0.04)	< 0.001
HAZ§								
Not stunted	Reference							
Stunted	−0.14 (−0.26, −0.02)	0.021						
BMIZ§								
Not thin	Reference							
Thin	−0.22 (−0.43, −0.04)	0.027						
Anemia status								
Not anemic							Reference	
Anemic							0.12 (0.01, 0.21)	0.024
Household level								
Number of people in house‡					−0.03 (−0.05, −0.00)	0.030		
Number of children in house‡			−0.04 (−0.06, −0.01)	0.003				

CI = confidence interval; BMIZ = body mass index for age; HA = height for age; SD = standard deviation.

95% CI are obtained from bias-corrected bootstrap analyses using 2,000 bootstrap samples.

* Scores standardized separately by class for each of the three domains (attention, numeracy, and literacy), so as to control for the effect of class and test. Differences are presented as z-scores from the class mean.

† *P* value is from the Wald test using multilevel linear regression models (accounting for school-level clustering).

‡ Modeled as a continuous variable.

§ Stunted and thin defined as HAZ and BMIZ z-scores < 2 SD.
